# Ability of Procalcitonin and C-Reactive Protein for Discriminating between Bacterial and Enteroviral Meningitis in Children Using Decision Tree

**DOI:** 10.1155/2021/5519436

**Published:** 2021-08-02

**Authors:** Dmitriy Babenko, Aliya Seidullayeva, Dinagul Bayesheva, Bayan Turdalina, Baurzhan Omarkulov, Aigul Almabayeva, Marina Zhanaliyeva, Almagul Kushugulova, Samat Kozhakhmetov

**Affiliations:** ^1^Karagandy Medical University, Karagandy, Kazakhstan; ^2^Innovative Center ArtScience, Nur-Sultan, Kazakhstan; ^3^Department of Paediatric Infectious Diseases, Astana Medical University, Nur-Sultan City, Kazakhstan; ^4^Department of Paediatric Infectious Diseases, №3 Multidisciplinary City Children's Hospital, Nur-Sultan City, Kazakhstan; ^5^NSC Astana Medical University, Nur-Sultan City, Kazakhstan; ^6^Centre for Life Sciences, National Laboratory Astana, Nazarbayev University, Nur-Sultan City, Kazakhstan

## Abstract

Bacterial meningitis (BM) is a public health burden in developing countries, including Central Asia. This disease is characterized by a high mortality rate and serious neurological complications. Delay with the start of adequate therapy is associated with an increase in mortality for patients with acute bacterial meningitis. Cerebrospinal fluid culture, as a gold standard in bacterial meningitis diagnosis, is time-consuming with modest sensitivity, and this is unsuitable for timely decision-making. It has been shown that bacterial meningitis differentiation from viral meningitis could be done through different parameters such as clinical signs and symptoms, laboratory values, such as PCR, including blood and cerebrospinal fluid (CSF) analysis. In this study, we proposed the method for distinguishing the bacterial form of meningitis from enteroviral one. The method is based on the machine learning process deriving making decision rules. The proposed fast-and-frugal trees (FFTree) decision tree approach showed an ability to determine procalcitonin and C-reactive protein (CRP) with cut-off values for distinguishing between bacterial and enteroviral meningitis (EVM) in children. Such a method demonstrated 100% sensitivity, 96% specificity, and 98% accuracy in the differentiation of all cases of bacterial meningitis in this study. These findings and proposed method may be useful for clinicians to facilitate the decision-making process and optimize the diagnostics of meningitis.

## 1. Introduction

Meningitis is a life-threatening inflammatory disease of the brain and spinal cord, mostly caused by bacterial, viral, and fungal infection [1–3]. Meningococcal infection has been a big threat to the globe and exists as a sporadic, hypersporadic, and epidemic disease. In 2012, an estimated 1.2 million cases of meningococcal infection per year were reported, with ~135,000 deaths worldwide [4]. The average annual incidence of meningococcal infection in Kazakhstan for the last decade is 0.83/100 000 with a peak in 2015 (2.42/100 000) [5].

Bacterial meningitis as a more serious form of meningitis is caused by pyogenic bacteria, such as *S. pneumoniae*, *N. meningitidis*, and *H. influenzae* [6]. Viruses are the most common cause of aseptic meningitis, primarily enteroviruses, together with numerous nonviral and noninfectious disorders [7, 8].

Although bacterial meningitis has a lower incidence rate than viral/aseptic meningitis [9, 10], prompt correct diagnosis and adequate treatment are necessary due to its hazardous nature [11]. Delay in the start of proper therapy introduces the potential for increased morbidity and mortality if the patient does indeed have acute bacterial meningitis [12].

Diagnosis of bacterial meningitis is based on a positive culture of cerebrospinal fluid (or detecting etiological agent by polymerase chain reaction—PCR), along with typical clinical symptoms (fever, headache, and neck stiffness). CSF culture is highly specific but lacks sensitivity, especially when antimicrobials have been given as well as the time needed until results appear [13]. In this case, PCR analysis can play a diagnostic role, but as the direct culture of cerebrospinal fluid, it takes some time. It should also be noted that not every clinic has the appropriate equipment and capabilities for conducting PCR analysis in CSF, especially in developing countries [14].

Distinguishing bacterial meningitis is often difficult [15] and therefore highly accurate decision support tools are necessary to guide decision making and limit unnecessary hospital admissions and prolonged antibiotic use.

Our study is aimed at assessing the role of clinical presentations, serum, and CSF profiles to distinguish BM and EVM in children.

## 2. Materials and Methods

### 2.1. Subjects

Recruiting patients for the study was carried out in the Department of Reanimation and Intensive Care, Infection Department No. 1, Multidisciplinary City Children's Hospital No. 3, Nur-Sultan City (Kazakhstan). The study covers the period between 2017 and 2019.

Inclusion criteria were as follows: children from 1 month to 17 years old, both sexes, presence of bacterial antigen, bacterial or viral nucleic acids identified in CSF in blood serum, results of a positive culture study for pathogens, and the presence of clinical signs of meningitis.

Exclusion criteria were as follows: children diagnosed with tuberculous meningitis, benign and malignant brain tumors, and children over 17 years old. The study did not include samples with meningitis of nonenteroviral etiology and combined forms of meningitis, bacteremia (meningococcemia).

The study has been approved by the Local Ethics Committee of the National Laboratory Astana (NLA) at Nazarbayev University (by 22^nd^ of September 2017. Approval No. 20).

A total of 269 patients were recruited and divided into 6 groups from 1 month to 10 years and more (Table 1).

### 2.2. Physical Examination

Clinical symptoms such as temperature, vomiting, impaired consciousness, headache, pallor of the skin, rash, tension, and bulging of the fontanel in children under one-year-old, stiff neck muscles, Lesage, Brudzinski, and Kernig symptoms were determined.

### 2.3. Laboratory Examination

The number of white blood cells (WBC), neutrophils, and level of protein in the CSF were determined using the Cobas Integra 400 plus analyzer (Roche, EU). The level of glucose in CSF was determined using the ABL800 Flex Analyzer (Radiometer Medical ApS, Denmark). The levels of haemoglobin, erythrocyte sedimentation rate (ESR), white blood cells, count, neutrophils, and platelets in blood were determined using the hematologic analyzer Sysmex XP-300 (Sysmex). The levels of CRP and procalcitonin were analysed using fluorescence immune-chromatographic system Finecare FIA Meter (Guangzhou Wondfo Biotech Co. Ltd., China.).

Samples of CSF or blood were placed on the surface of the culture medium for cultivation and identification on the “chocolate” agar (based on trypticase soy agar with the addition of defibrinated ram blood). Then, the samples were incubated at a temperature of 37.0°C in an atmosphere of 5.0% CO_2_ for 24-48 hours. The presence of etiological agents of viral meningitis was determined by commercial PCR kits according to the manufacturer's protocol.

### 2.4. Statistical Analysis

Statistical analysis was carried out using SigmaPlot 11.0 software (Systat Software Inc., USA) with the following conditions:

1. *Quantitative Data with a Normal Distribution*. Student *t*-test for two groups or analysis of variance (ANOVA), when the number of groups is more than two
2. *Quantitative Data with Abnormal Distribution*. Nonparametric Kruskal-Wallis test and Mann–Whitney test for independent groups; and Wilcoxon Matched Pairs Test for dependent groups
3. *Categorical Data*. Chi-square or Fisher's tests if necessary (when the expected frequency is less than 5 in one of the cells)

Shapiro-Wilk test was employed for the evaluation of the distribution of data (normality test). *p* < 0.05 was considered statistically significant for all analyses.

### 2.5. Modelling

Fast-and-frugal trees (FFTs) as a supervised learning algorithm described in Phillips, Neth, Woike, and Gaissmaier [16] and implemented in FFTrees R package was used to predict a binary criterion BM and EVM. Before the machine training, we split the entire dataset into training (80%) and testing (20%) subsets. An optimal cut-off point was calculated according to the highest accuracy (minimal false-negative and false-positive results). The area (AUC, area under curve) under the receiver operating characteristic curve (ROC) was used to check the prognostic value of a particular parameter. Sensitivity, specificity, positive predictive value (PPV), negative predictive value (NPV), and accuracy were calculated for the given cut-off values for predicting bacterial meningitis.

## 3. Results

A total of 269 children (117 females and 152 males) were included in this study. The median and IQR age of the participants were 48.0 [17.0-96.0] months old. Children with EVM, that had a median age of 82.5 [40.0; 124] months old, were older compared with the BM group (median age 23.0 [9.00; 50.0]; *p* < 0.001) (Table 1). The highest rate of meningitis was found out among children aged up to 1-year old in a group with BM. In contrast, the rate of EVM was relatively low (7.53%) in a group up to 1-year children, while in groups > 1 year, EVM incidences were higher (on average18.48%) and reached 26% in the group > 10 years.

Among the studied patients, bacterial meningitis represented 45.7% (123 patients) compared to 54.3% (146 patients) nonbacterial (enteroviral) meningitis. Among bacterial meningitis, 95 were due to *N. meningitidis*, 25 cases *S. pneumoniae*, 2 cases *S. agalactiae*, and 1 case *S. aureus*. Among 146 viral/aseptic cases, all 146 cases were caused by enterovirus.

The most common presenting symptom in children with BM was the high temperature (39°C vs. 37.9°C, *p* < 0.001) followed by headache (82.9%) and Kernig's sign (82.1%). The occurrence of Brudzinski's sign (44.7%), drowsiness (25.2%), spasms (22%), and loss of consciousness (12.2%) was significantly higher in the BM group compared with the EVM group (*p* < 0.001). Vomiting, bulging fontanelle, and neck rigidity had no difference among these groups. The clinical features of BM and EVM groups are presented in Table 2.

Table 3 represents the comparison of the laboratory results of blood and CSF between the two groups. Blood and CSF laboratory testing data showed significantly increased levels of proteins, CRP, procalcitonin, WBC, neutrophils, and platelets in the blood of children with BM (*p* < 0.001). WBC and neutrophils in CFS were also significantly higher in the BM group (*p* < 0.001). The blood glucose level was 1.50 [0.57; 1.90] mmol/L in the BM group, which is significantly lower than that for the EVM group (3.70 [3.10; 4.80] mmol/L; *p* < 0.001). Haemoglobin was also significantly lower in the BM group in comparison with the EVM group (*p* < 0.001).

**Figure 1:**
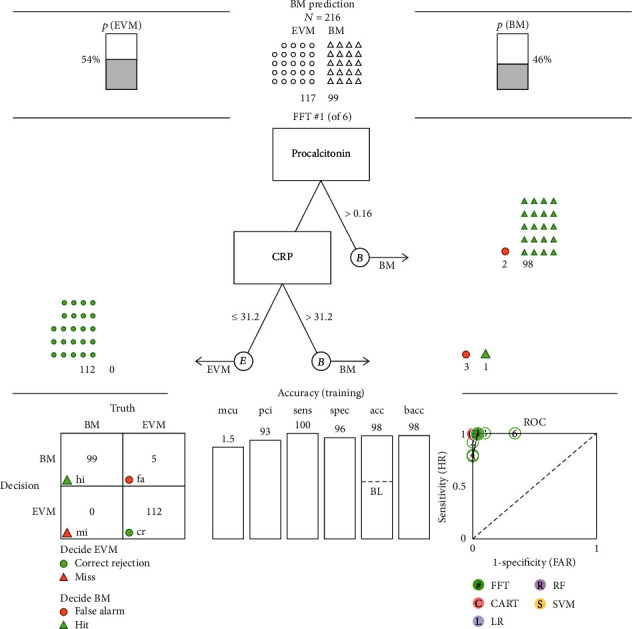
A fast-and-frugal tree (FFT) for classifying patients as either with BM or with EVM based on up to two parameters. It should be interpreted as if a patient's procalcitonin value is more than 0.16 ng/mL, classify as BM. If not, check the CRP value. If this is less or equal 31.2 mg/L, classify as EVM, otherwise, classify as BM.

Fast-and-frugal trees (FFTs) algorithm was performed for providing efficient and accurate decisions in the prediction of bacterial meningitis. All data, such as demographic variables (gender and age), clinical data, and laboratory results, were included in training FFTs to get the best-trained algorithm based on the highest sensitivity. The results of the best FFT with sensitivity, specificity, accuracy, and ROC curve are presented in Figure 1.

Two parameters, procalcitonin and C-reactive protein, appeared to have a good predictive value in bacterial meningitis. ROC curve was plotted with these two parameters having the best performing curve for diagnosing bacterial meningitis with a sensitivity of 100%, a specificity of 96%, and an accuracy of 98%.

## 4. Discussion

Meningococcal infection is one of the most severe infectious diseases of childhood [11]. The highest burden of disease is in Africa and Asia. However, the epidemics can occur in any part of the world. According to WHO reports, Asia has had some major epidemics of meningococcal disease in the last 30 years [17–20]. According to official statistical data, the peak of meningitis incidence in Kazakhstan was noted in 2015 (2.4 per 100 000). In subsequent years, there was a trend towards a decrease in 3.6 times by 2016, 7 times by 2017, and 4.6 times by 2018 compared to 2015 [5].

The early diagnostics of meningitis and differentiation of bacterial forms from aseptic (viral) ones plays a crucial role in the effective treatment of children. The analysis of CSF culture, as a gold standard in bacterial meningitis diagnostics, is a time-consuming process with modest sensitivity (70–85%). Moreover, in the case of antibiotic pretreatment, the sensitivity of CSF culture decreases by 20% [21]. In this regard, the CFS culture test is unsuitable for timely decision-making and effective diagnostics. The classic approach for the differentiation between bacterial and viral meningitis is based on the assessment of clinical signs and symptoms and laboratory tests (blood and CSF analysis) [11, 22, 23].

In contrast to the standard method, our study is focused on searching a novel method of meningitis diagnostics and differentiation. The proposed method is based on the validation of the factors such as demographic variables, clinical, and routine diagnostic tests, with the most discriminating power and sensitivity in differentiating bacterial meningitis from nonbacterial (enteroviral) meningitis.

Our trained FFTree model determined two parameters, procalcitonin and CRP in blood, based on which the differentiation of BM from EVM is effective. Definition of the decision tree is “if procalcitonin > 0.16 ng/mL, decide BM, If CRP < = 31.2 mg/L, decide EVM, otherwise, decide BM”. This trained FFT model demonstrated an extremely high sensitivity (100% vs. 100%) and NPV (100% vs. 100%), and very high specificity (95.7% vs. 93%), PPV (95% vs. 92.3%), and accuracy (97.7% vs. 96.2%) on both trained and test datasets, respectively. The parameters of the predictive ability of each indicator are shown in Table 4.

The results demonstrated that the top three parameters (procalcitonin, CRP, and glucose level) were of the highest accuracy (98.6%, 95.8%, and 90.3%, respectively) in discriminating between bacterial and enteroviral meningitis. Previous studies also showed the same results with variation in cut-off values to distinguish bacterial and viral meningitis [24–31]. At the same time, the authors emphasized the importance of the heterogeneity of populations, techniques, and approaches of decision-making threshold for BM diagnosis markers.

In this paper, we addressed the task of distinguishing bacterial from viral meningitis in children through a machine learning-based approach deriving making decision rules. The proposed FFTree decision tree approach showed an ability to determine procalcitonin and CRP in blood with cut-off values for distinguishing between bacterial and enteroviral meningitis in children. It should be noted that the proposed method uses a minimally invasive procedure for taking material for diagnosis. Also, the method demonstrated 100% sensitivity, 96% specificity, and 98% accuracy in differentiation of all cases of bacterial meningitis in this study. These findings and proposed method may be useful for clinicians to facilitate the decision-making process and optimize the diagnostics of meningitis.

## Figures and Tables

**Table 1:** Demographic data of the studied population with meningitis.

	[ALL]	EVM	BM	*p*.overall
	*N* = 269	*N* = 146	*N* = 123	
Age (in months)	48.0 [17.0; 96.0]	82.5 [40.0; 124]	23.0 [9.00; 50.0]	<0.001
Age group:				<0.001
≤1 y	52 (19.3%)	11 (7.53%)	41 (33.3%)	
>1-3 y	61 (22.7%)	23 (15.8%)	38 (30.9%)	
>3-5 y	46 (17.1%)	27 (18.5%)	19 (15.4%)	
>5-7 y	33 (12.3%)	17 (11.6%)	16 (13.0%)	
>7-10 y	36 (13.4%)	30 (20.5%)	6 (4.88%)	
>10 y	41 (15.2%)	38 (26.0%)	3 (2.44%)	
Gender:				0.622
Female	117 (43.5%)	66 (45.2%)	51 (41.5%)	
Male	152 (56.5%)	80 (54.8%)	72 (58.5%)	

**Table 2:** Underlying and associated conditions in bacterial and enteroviral meningitis groups in the studied population.

	[ALL]	EVM	BM	*p*.overall
	*N* = 269	*N* = 146	*N* = 123	
Temperature (^o^C):	38.5 [37.8; 39.0]	37.9 [37.4; 38.5]	39.0 [38.6; 39.5]	<0.001
Vomiting:	262 (97.4%)	144 (98.6%)	118 (95.9%)	0.252
Headache:	146 (54.3%)	44 (30.1%)	102 (82.9%)	<0.001
Bulging fontanelle (for ≤18 months olds):	71 out 72 (98.6%)	19 out 20 (95%)	52 out 52 (100%)	0.278
Neck rigidity:	266 (98.9%)	146 (100%)	120 (97.6%)	0.094
Kernig's sign:	168 (62.5%)	67 (45.9%)	101 (82.1%)	<0.001
Brudzinski's sign:	61 (22.7%)	6 (4.11%)	55 (44.7%)	<0.001
Loss of consciousness:	15 (5.58%)	0 (0.00%)	15 (12.2%)	<0.001
Drowsiness:	36 (13.4%)	5 (3.42%)	31 (25.2%)	<0.001
Spasms:	27 (10.0%)	0 (0.00%)	27 (22.0%)	<0.001

**Table 3:** Laboratory findings of blood and cerebrospinal fluid in the studied population.

	[ALL]	EVM	BM	*p*.overall
	*N* = 269	*N* = 146	*N* = 123	
Glucose in CSF: [2.3-3.9] mmol/L	2.80 [1.60; 3.80]	3.70 [3.10; 4.80]	1.50 [0.57; 1.90]	<0.001
Haemoglobin in blood: [110-140] g/L	119 (16.7)	125 (15.4)	111 (15.2)	<0.001
Protein in CSF: [0.12-0.45] g/L	0.50 [0.20; 1.30]	0.20 [0.10; 0.40]	1.50 [0.60; 2.10]	<0.001
CRP in blood: [≤10] mg/L	22.8 [4.80; 110]	5.00 [2.12; 12.0]	118 [43.5; 196]	<0.001
ESR in blood: [0-10] mm/H	15.0 [10.0; 22.0]	12.0 [7.00; 17.0]	18.0 [14.5; 30.0]	<0.001
Procalcitonin in blood: [≤0.05] ng/mL	0.05 [0.02; 3.30]	0.02 [0.01; 0.03]	3.50 [2.15; 5.15]	<0.001
WBC in CSF: [≤30]∗10^9^/L	380 [110; 1300]	126 [78.2; 233]	1455 [815; 4250]	<0.001
WBC in blood: [4.5 − 10.5]∗10^9^/L	13.1 [8.80; 18.0]	9.25 [7.60; 12.2]	18.0 [15.0; 23.2]	<0.001
Neutrophils in CSF: [≤10]∗10^6^/L	72.0 [19.0; 90.0]	21.5 [10.0; 61.5]	88.0 [75.0; 90.0]	<0.001
Neutrophils in blood: [42-72%]	79.8 [69.9; 87.1]	74.0 [58.2; 84.0]	85.0 [76.3; 90.0]	<0.001
Platelets in blood: [180 − 320]∗10^9^/L	233 [195; 314]	228 [190; 288]	256 [209; 321]	0.020

**Table 4:** Validation of clinical and laboratory parameters in bacterial meningitis prediction.

Parameters	Threshold	Direction	*N*	Sens	Spec	PPV	NPV	Acc
Procalcitonin in blood (ng/mL)	0.16	>	216	0.990	0.983	0.980	0.991	0.986
CRP in blood (mg/L)	31.2	>	216	0.939	0.974	0.969	0.950	0.958
Glucose in CSF (mmol/L)	2.2	<=	216	0.848	0.949	0.933	0.881	0.903
Neutrophils in CSF (∗10^6^/L)	64	>	216	0.980	0.726	0.752	0.977	0.843
WBC in CSF (∗10^9^/L)	513	>	216	0.818	0.872	0.844	0.850	0.847
Temperature (°C)	38.4	>	216	0.909	0.752	0.756	0.907	0.824
Protein in CSF (g/L)	0.3	>	216	0.949	0.709	0.734	0.943	0.819
WBC in blood (∗10^9^/L)	12.5	>	216	0.879	0.769	0.763	0.882	0.819
Headache	Yes	=	216	0.838	0.701	0.703	0.837	0.764
Age (months)	85	≤	216	0.949	0.470	0.603	0.917	0.690
Kernig's sign	Positive	=	216	0.838	0.538	0.606	0.797	0.676
Brudzinski's sign	Positive	=	216	0.424	0.949	0.875	0.661	0.708
Neutrophils in blood (%)	73	>	216	0.879	0.453	0.576	0.815	0.648
ESR in blood (mm/H)	11	>	216	0.859	0.462	0.574	0.794	0.644
Haemoglobin in blood (g/L)	113	≤	216	0.576	0.744	0.655	0.674	0.667
Spasms	Single	=	216	0.212	1.000	1.000	0.600	0.639
Drowsiness	Yes	=	216	0.232	0.957	0.821	0.596	0.625
Platelets in blood (∗10^9^/L)	252	>	216	0.545	0.607	0.540	0.612	0.579
Consciousness	Nonnormal	=	216	0.111	1.000	1.000	0.571	0.593
Gender	M	=	216	0.566	0.444	0.463	0.547	0.500

Abbreviations: sens: sensitivity; spec: specificity; ppv: positive predictive value; npv: negative predictive value; acc: accuracy.

## Data Availability

The datasets used and/or analysed during the current study are available from the corresponding author on reasonable request.
